# Mitochondrial diseases caused by toxic compound accumulation: from etiopathology to therapeutic approaches

**DOI:** 10.15252/emmm.201505040

**Published:** 2015-07-20

**Authors:** Ivano Di Meo, Costanza Lamperti, Valeria Tiranti

**Affiliations:** Unit of Molecular Neurogenetics, Foundation IRCCS Neurological Institute C. BestaMilan, Italy

**Keywords:** mitochondrial diseases, OXPHOS, sulfide catabolism, therapeutic approaches, thymidine/deoxyuridine catabolism

## Abstract

Mitochondrial disorders are a group of highly invalidating human conditions for which effective treatment is currently unavailable and characterized by faulty energy supply due to defective oxidative phosphorylation (OXPHOS). Given the complexity of mitochondrial genetics and biochemistry, mitochondrial inherited diseases may present with a vast range of symptoms, organ involvement, severity, age of onset, and outcome. Despite the wide spectrum of clinical signs and biochemical underpinnings of this group of dis-orders, some common traits can be identified, based on both pathogenic mechanisms and potential therapeutic approaches. Here, we will review two peculiar mitochondrial disorders, ethylmalonic encephalopathy (EE) and mitochondrial neurogastrointestinal encephalomyopathy (MNGIE), caused by mutations in the *ETHE1* and *TYMP* nuclear genes, respectively. *ETHE1* encodes for a mitochondrial enzyme involved in sulfide detoxification and *TYMP* for a cytosolic enzyme involved in the thymidine/deoxyuridine catabolic pathway. We will discuss these two clinical entities as a paradigm of mitochondrial diseases caused by the accumulation of compounds normally present in traces, which exerts a toxic and inhibitory effect on the OXPHOS system.

See also: **A Suomalainen** (October 2015)

## Introduction

Mitochondria are the main source of adenosine triphosphate (ATP), which is synthesized by the mitochondrial respiratory chain (MRC) through the oxidative phosphorylation (OXPHOS) process. ATP is the main energy substrate required for all active cellular processes, and its deficiency leads to cellular dysfunction and ultimately cell death. Energy failure due to reduced ATP biosynthesis appears to be critical in individual or combined MRC complex defects, but other mechanisms are also likely to be involved, and might be even predominant in the pathogenesis of specific syndromes, including alterations of cellular reduction–oxidation status, compromised Ca^2+^ homeostasis, dysregulation of mitochondrial dynamics, autophagy or apoptosis, in addition to the production of reactive oxygen species (ROS) or accumulation of toxic metabolism intermediates (Wallace, 2005).

Although at a first glance ethylmalonic encephalopathy (EE) and mitochondrial neurogastrointestinal encephalomyopathy (MNGIE) may appear to be unrelated diseases, they actually share an unconventional pathogenic mechanism, the accumulation of toxic compounds such as sulfide and deoxyribonucleoside, respectively, which determine secondary OXPHOS deficiency. Specifically, while in EE, the accumulation of sulfide causes the inhibition of cytochrome c oxidase (COX), the systemic accumulation of thymidine and deoxyuridine in MNGIE determines an imbalance of the dNTP pool that interferes with mitochondrial DNA (mtDNA) replication and leads to depletion, multiple deletions and point mutations, and thus mitochondrial dysfunction.

## Ethylmalonic encephalopathy

EE (OMIM #602473), first described by Burlina *et al* (1991), is an autosomal recessive, invariably fatal mitochondrial disorder characterized by early-onset brain failure, vascular lesions producing petechial purpura and orthostatic acrocyanosis, and chronic hemorrhagic diarrhea. The onset and degree of severity of these symptoms vary from patient to patient but usually occur early in development. Brain MRI reveals bilateral lesions in the basal nuclei and brainstem gray matter inducing the typical neurological symptoms of the disease, namely psychomotor regression, dystonia, axial hypotonia, spastic tetraparesis, and seizures.

In addition, patients also exhibit several biochemical traits including a typical albeit unusual combination of severe deficiency of the terminal component of the mitochondrial respiratory chain cytochrome c oxidase (COX) in muscle, brain, and colonic mucosa, leading to high levels of lactate in blood, and accumulation of ethylmalonic acid (EMA) and C4- and C5-acylcarnitines in fluids. These are typically found in the presence of defects of the short-chain and branched-chain acyl-CoA dehydrogenases (SCAD, BCAD); SCAD and BCAD activities, however, are normal in EE fibroblasts and muscle (Nowaczyk *et al*, 1998).

In 2004, the EE locus was identified by autozygosity mapping in a region of chromosome 19, demonstrating the presence of a cohort of pathogenic mutations in the responsible gene, termed *ETHE1*, for ethylmalonic encephalopathy 1 (Tiranti *et al*, 2004). Mutations in the *ETHE1* gene have been identified in more than 80 EE patients worldwide (Tiranti *et al*, 2004, 2006; Mineri *et al*, 2008; Drousiotou *et al*, 2011; Tiranti & Zeviani, 2013). Despite most of the changes found in *ETHE1* gene cause protein loss, some of the missense mutations are associated with its presence in patient fibroblasts and/or muscle biopsies, suggesting that the corresponding wild-type amino-acid residues have a catalytic function (Tiranti *et al*, 2006). The availability of the crystal structure of human ETHE1 (Pettinati *et al*, 2015) now makes it possible to analyze the spatial location of the amino acid changes predicted by missense mutations and to classify them as catalytic versus structural.

ETHE1 is a 30-kDa polypeptide exclusively located in the mitochondrial matrix, which functions as a homodimeric, Fe-containing sulfur dioxygenase (SDO) (Tiranti *et al*, 2009) involved in the catabolic oxidation of hydrogen sulfide (sulfide, H_2_S) to sulfate. Impairment of ETHE1 activity causes chronic accumulation of sulfide in critical tissues, including colonic mucosa, liver, muscle, and brain, in both humans and an *Ethe1*^−/−^ mouse model (Tiranti *et al*, 2009).

## Hydrogen sulfide in health and disease

H_2_S is a colorless, water-soluble gas with a typical odor of rotten eggs. Although in the past it has been investigated as a toxic gas, it is now recognized as a physiologically relevant gasotransmitter, which can easily penetrate the plasma membranes of cells due to its high solubility in lipids.

**Figure 1:**
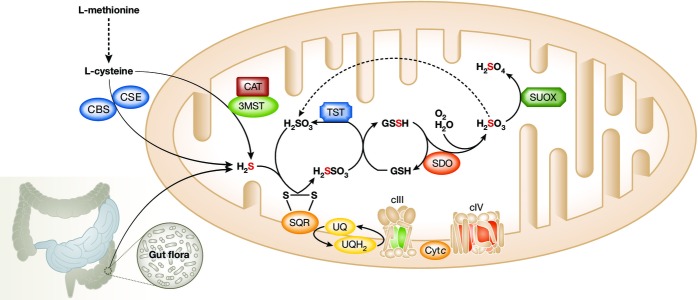
Endogenous sulfide metabolism H_2_S is produced endogenously from cysteine by cystathionine β-synthase (CBS), cystathionine γ-lyase (CSE), cysteine aminotransferase (CAT), and 3-mercaptopyruvate sulfurtransferase (3-MST), or exogenously by gut anaerobic bacterial flora metabolism. In mitochondria, it is initially fixed by a sulfide quinone oxidoreductase (SQR). A sulfur dioxygenase (SDO/ETHE1) oxidizes the persulfide to sulfite (H_2_SO_3_) in a reaction that includes molecular oxygen and water. See text for details. SUOX, sulfite oxidase; TST, thiosulfate sulfur transferase; cIII, complex III; cIV, complex IV.

Hydrogen sulfide is endogenously produced in mammals by cystathionine β-synthase (CBS) and cystathionine γ-lyase (CSE), and by tandem catalysis by cysteine aminotransferase (CAT) and 3-mercaptopyruvate sulfurtransferase (3-MST) (Fig 1). CBS, CSE, and CAT/3-MST are cytosolic enzymes, whereas CAT and 3-MST are also present in mitochondria (Kamoun, 2004). All the above enzymes utilize as a substrate L-cysteine (Abe & Kimura, 1996) that can be taken up with the diet, extracted from endogenous proteins, or synthesized endogenously via trans-sulfuration of serine by L-methionine.

In addition to its endogenous production, a substantial amount of H_2_S derives from the intestinal anaerobic bacterial flora residing in the intestinal lumen (Fig 1). The intestinal epithelium expresses specialized enzyme systems that efficiently convert H_2_S into thiosulfate and sulfate, to prevent the local increase in H_2_S to toxic levels and its entry through the portal vein system in the liver and other organs.

The main catabolic pathway of H_2_S takes place in mitochondria, and although the existence of such a pathway has been known for more than 20 years (Powell & Somero, 1986), the enzymatic actors and detailed steps have been elucidated (Hildebrandt & Grieshaber, 2008) and further refined (Jackson *et al*, 2012) only recently. The pathway consists of (1) mitochondrial inner-membrane-bound sulfide quinone reductase (SQR), which fixes H_2_S to sulfite (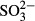) to produce thiosulfate (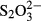); (2) mitochondrial thiosulfate sulfur transferase (TST), also known as rhodanese, which reconstitutes sulfite from thiosulfate by fixating the sulfane sulfur of the latter onto an –SH-containing substrate, for instance, glutathione, GSH, to form a persulfide (R-S-SH) species; (3) mitochondrial matrix sulfur dioxygenase (ETHE1-SDO), which oxidizes the sulfur atom extracted from persulfide, converting it again into sulfite; and (4) mitochondrial sulfite oxidase (SUOX), which further oxidizes sulfite into sulfate (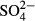) (Fig 1).

**Figure 2:**
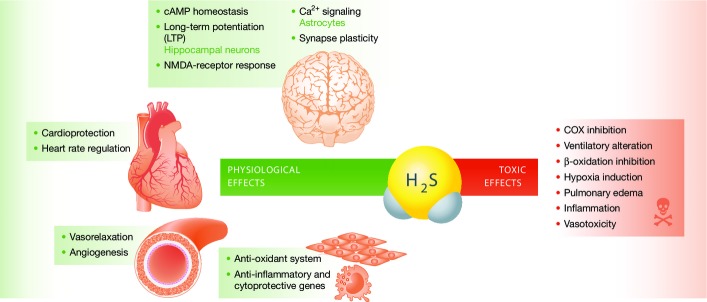
H_2_S in health and disease Physiological (green bordered) and toxic (red bordered) effects of H_2_S in mammal tissues and organs. See text for details.

H_2_S has many physiological functions (Fig 2). In the CNS, it increases the production of cAMP, enhances N-methyl-d-aspartate (NMDA)-receptor mediated responses, and facilitates the induction of long-term potentiation (LTP) in hippocampal neurons (Kimura & Kimura, 2004). In astrocytes, H_2_S induces Ca^2+^ influx that propagates to the surrounding astrocytes in the form of Ca^2+^ waves (Nagai *et al*, 2004). H_2_S also acts on active but not quiescent synapses, suggesting an involvement in associative learning (Abe & Kimura, 1996).

In myocardial and vascular smooth muscle cells, through the activation of ATP-dependent K^+^ (K_ATP_) channels, H_2_S depresses the activity of the sinoatrial pacemaker, thus reducing heart rate (Xu *et al*, 2008), and hyperpolarizes membranes, causing vasorelaxation (Elsey *et al*, 2010). In addition, H_2_S inhibits phosphodiesterases (Bucci *et al*, 2010), which also promotes vasorelaxation. Another effect is the induction of smooth muscle cell proliferation by the activation of the PI3 Kinase/Akt pathway, thus stimulating angiogenesis and apoptosis via the MAPK pathway (Yang *et al*, 2004).

Furthermore, H_2_S induces upregulation of antioxidant systems (Whiteman *et al*, 2004) and of anti-inflammatory and cytoprotective genes including heme oxygenase (HO1) in pulmonary smooth muscle cells (Qingyou *et al*, 2004), and macrophages (Oh *et al*, 2006). H_2_S exerts a protective role during ischemia/reperfusion injury. During ischemia, cells are in hypoxic conditions, and subsequent reperfusion and reoxygenation leads to tissue damage caused by increasing reactive oxygen species (ROS) production, inflammation, and cell death, rather than restoration of normal functions. The protective effects of H_2_S are mediated by the activation of the cardioprotective PI3K/Akt pathway and of the KATP channels, and increase in the free thiol pool, which acts as a ROS scavenger (Ferdinandy *et al*, 2007 ).

Although H_2_S acts as a cytoprotective agent at physiological (nanomolar) concentrations, at micromolar concentrations, it can interfere with a variety of cellular functions (Fig 2), including mitochondrial respiration via inhibition of COX (Hill *et al*, 1984) by the formation of a covalent bond between with the Fe atom coordinated by heme a. The chronic exposure and binding of H_2_S to COX causes accelerated degradation of its protein subunits (Di Meo *et al*, 2011). H_2_S is also an inhibitor of carbonic anhydrase (Coleman, 1967), which could explain the alterations of ventilatory dynamics in response to inhaled H_2_S (Klentz & Fedde, 1978). A further effect of H_2_S is inhibition of enzymatic activity of SCAD, which can cause dicarboxylic aciduria (Pedersen *et al*, 2003). Acute inhalation of H_2_S causes anoxic brain injury, pulmonary edema, and death (Yalamanchili & Smith, 2008).

## Sulfide toxicity in EE

Impaired activity of ETHE1-SDO in EE leads to the accumulation of H_2_S in critical tissues, including colonic mucosa, liver, muscle, and brain, up to concentrations that inhibit SCAD and COX activities, thus inducing EMA aciduria, and high levels of C4- and C5-acylcarnitines, EMA, and lactate in plasma (Tiranti *et al*, 2009). Chronic COX inhibition by accumulated H_2_S leads to accelerated degradation of the COX protein backbone (Di Meo *et al*, 2011). Interestingly, although ETHE1 activity is absent in all organs of *Ethe1*^−/−^ mice, the muscle, brain, and colon feature both defective COX activity and reduced COX levels, while the liver appears normal. This may reflect the presence of alternative metabolic pathways protecting hepatocytes from H_2_S toxicity.

Dietary restriction in rodents stimulates the CSE expression and results in increased H_2_S production, even if not comparable to those caused by *ETHE1* mutations, determining functional benefits such as protection from ischemia reperfusion injury (Hine *et al*, 2015). Although these findings are apparently in contrast to the detrimental effects of excessive concentrations of H_2_S in EE, the H_2_S-induced functional benefits might require an intact MRC coupled with SQR, potentially serving as a source of electrons during ischemia (Hine *et al*, 2015). Hence, since MRC is compromised in EE due to COX degradation, H_2_S would not be able to induce the beneficial effects described above.

In addition to COX and SCAD deficiency, other EE signs are explained by the accumulation of H_2_S, including damage of endothelial cells, and vasodilation, which account for the petechiae and the acrocyanosis. Analysis of autoptic specimens in a genetically confirmed human patient showed morphologic evidence of diffuse vascular damage (Giordano *et al*, 2012). Analysis of the brain showed widespread luminal microthrombi, acute microhemorrhages and focal perivascular hemosiderinladen macrophages. Endothelial damage and loss were observed also in the gastric antrum and colonic mucosa and submucosa, associated with microhemorrhages. Interestingly, histological examination of brain and colonic mucosa of the *Ethe1*^−/−^ mouse also revealed diffuse vascular damage. Since a substantial amount of H_2_S derives from the anaerobic bacterial flora residing in the intestinal lumen, COX activity is markedly reduced in the luminal colonocytes of *Ethe1*^−/−^ mice, while it is normal in cryptal colonocytes that are relatively protected from the luminal surface. This disparity is thus likely due to the different exposure of the two cell populations to the inhibitory action of exogenous H_2_S. Excessive production and absorption of H_2_S, as well as reduced detoxification by colonocytes, is considered to play an important role in the mucosal damage of ulcerative colitis (Rowan *et al*, 2009). A similar mechanism may well account for the severe chronic diarrhea afflicting EE patients.

Proteomic analysis of *Ethe1*^−/−^ mice tissues provides evidence for a crucial role of the mitochondrial sulfide oxidation pathway in sulfide signaling. Increased sulfide concentrations lead to major disturbances in post-translational protein modifications (Hildebrandt *et al*, 2013). The mechanistic foundation of this regulatory function is most likely based on cysteine S-modifications, suggesting the involvement of sulfide in redox regulation, and cytoskeleton dynamics and regulation of mitochondrial fatty acid catabolism.

Muscle- or brain-restricted ablation of ETHE1 is clearly associated with an isolated COX deficiency in the targeted tissue but not in other, ETHE1 competent, tissues (Di Meo *et al*, 2011). These data unequivocally demonstrate that failure to neutralize the endogenous production of H_2_S is sufficient for COX activity to decrease, but not for the animals to become sick or for thiosulfate, a specific biomarker of the disease, to increase. This observation suggests that multiorgan accumulation of H_2_S and diffusion of exogenously released H_2_S from the bacterial flora are both needed to establish the severe metabolic impairment and the fatal clinical course of ETHE1-less mice and humans.

## Mitochondrial neurogastrointestinal encephalopathy

MNGIE (OMIM #603041) is an autosomal recessive disorder clinically defined by extraocular muscle weakness causing ptosis and ophthalmoplegia, peripheral neuropathy, severe gastrointestinal dysmotility, leukoencephalopathy, hearing loss, absent reflexes, and mitochondrial abnormalities including multiple deletions of mitochondrial DNA (mtDNA) in skeletal muscle (Nishino *et al*, 2000). Gastrointestinal dysmotility is the most prominent and debilitating clinical feature, can affect any portion of the enteric system and includes borborygmi, cachexia, diarrhea, early satiety, abdominal cramps, nausea, vomiting, intestinal pseudo-obstruction, and gastroparesis (Hirano *et al*, 1994). The disease is progressive and fatal with an average onset at 19 years of age and an average death at 37 years.

Biochemically, more than 50% of the patients present with lactic acidosis and morphologic evidence of abnormal mitochondria in muscle. COX deficiency has been observed in cultured skin fibroblasts from MNGIE patients and in other tissues (Hirano *et al*, 2004). Moreover, the analysis of mtDNA of muscle and other tissues has shown depletion and multiple deletions (Hirano *et al*, 1994), in addition to site-specific somatic point mutations in most tissues (Nishigaki *et al*, 2003). The somatic site-specific point mutations typical of MNGIE render this disease unique.

The first patient was reported in 1976, as a case of congenital oculoskeletal myopathy with abnormal muscle and liver mitochondria (Okamura *et al*, 1976). The disease locus was mapped in 1998 to chromosome 22, where mutations in the *ECGF1* gene (today known as *TYMP*) were identified as the culprits in MNGIE (Nishino *et al*, 1999). Since then, more than 140 additional individuals with MNGIE have been described (Hirano *et al*, 2012; Peedikayil *et al*, 2015). *TYMP* is expressed in most human tissues and organs, but with little or no expression in gallbladder, aorta, muscle, fat, and kidney (Matsukawa *et al*, 1996). It encodes for thymidine phosphorylase (TP), a 50-kDa cytosolic protein that functions *in vivo* as a homodimeric enzyme that catalyzes the phosphorolysis of deoxythymidine (dThd) or deoxyuridine (dUrd) to the corresponding base thymine or uracil, and 2-deoxy-D-ribose1-phosphate (Webster *et al*, 2001). Impairment of TP activity leads to systemic accumulation of the pyrimidine metabolism intermediates dThd and dUrd.

## Pyrimidine metabolism

Pyrimidines are aromatic heterocyclic organic compounds, and vital components of all living cells participating in a wide range of diverse biological functions. For instance, pyrimidines form the nucleotides that are vital building blocks for DNA (cytosine and thymine) and RNA (uracil and cytosine). In addition, they are also involved in a variety of fundamental cell functions, of which the most important are regulation of cell metabolism, energy conservation and transport, formation of coenzymes and of active intermediates of phospholipid and carbohydrate metabolism, glycosylation of proteins and lipids, functional vasoregulatory role through novel endothelium-derived vasoactive dinucleotides, and signal transduction and translation (Balasubramaniam *et al*, 2014). Pyrimidine nucleotides are either synthesized entirely within the body by a multistep (and energetically demanding) *de novo* synthesis pathway, by the recycling (via the salvage pathway) of nucleosides derived from catabolism during the normal process of cell turnover, or acquired from the diet (Berg *et al*, 2002).

**Figure 3:**
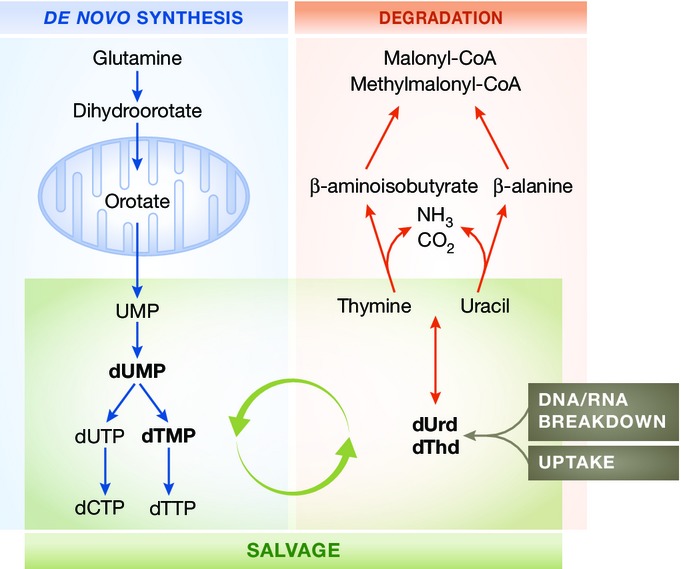
Summarized pyrimidines metabolism Interconnections between biosynthetic (*de novo* synthesis, blue bordered), catabolic (degradation, red bordered), and recycling (salvage, green bordered) pyrimidine pathways in mammalian cells. See text for details.

The biosynthetic *de novo* pathway occurs in six steps, with cellular compartmentalization of specific steps in the cytosol or mitochondria. Glutamine is first converted to dihydroorotate (DHO) through three sequential enzymatic reactions catalyzed by the trifunctional protein CAD (carbamoylphosphate synthetase/ATCase/dihydroorotase). Orotic acid is then formed by dihydro-orotate dehydrogenase (DHODH), located on the outer surface of the inner mitochondrial membrane, and functionally connected to the respiratory chain via ubiquinone. Orotate is then converted into the key nucleotide uridine-5′-monophosphate (UMP) by UMP synthase and then phosphorylated to UDP. Next, UDP can be reduced by ribonucleotide reductase (RNR) to dUMP that in turn can be phosphorylated to dUTP, or utilized as a substrate for CTP synthase for the synthesis of dCTP. Deoxyuridine nucleotides are also the precursors for *de novo* synthesis of the deoxythymidine nucleotides: Thymidylate synthase converts dUMP to dTMP, which can be phosphorylated to dTTP (Berg *et al*, 2002) (Fig 3). Of note, it has been recently found that in endothelial cells, fatty acid oxidation promotes *de novo* synthesis by providing carbons through the Krebs cycle, which are incorporated into aspartate and glutamate (nucleotide precursors), UMP and DNA (Schoors *et al*, 2015).

Pyrimidine catabolism occurs in liver, where unrecycled nucleosides are degraded to β-alanine (deoxycytidine monophosphate or uridine monophosphate) or β-aminoisobutyrate (deoxythymidine monophosphate), ammonia (NH_3_), and CO_2_. β-alanine and β-aminoisobutyrate serve as -NH_2_ donors in the transamination of α-ketoglutarate to glutamate. A subsequent reaction converts the products to malonyl-CoA, subsequently diverted to fatty acid synthesis, or methylmalonyl-CoA, which is converted to succinyl-CoA and can enter into the tricarboxylic acid (TCA) cycle (Berg *et al*, 2002) (Fig 3).

Finally, the salvage pathway is the contact point between the biosynthetic and degradative pathways and features a number of cytoplasmic or mitochondrial kinases that convert nucleosides generated by DNA and RNA breakdown back to nucleotide monophosphates, enabling reentry in the pyrimidine biosynthesis pathway (Berg *et al*, 2002) (Fig 3).

TP is the key enzyme in the above catabolic pathways, as it catalyzes the cleavage of deoxythymidine or deoxyuridine to thymine or uracil and ribose-1-phosphate.

## Deoxynucleotide pool imbalance in MNGIE

**Figure 4:**
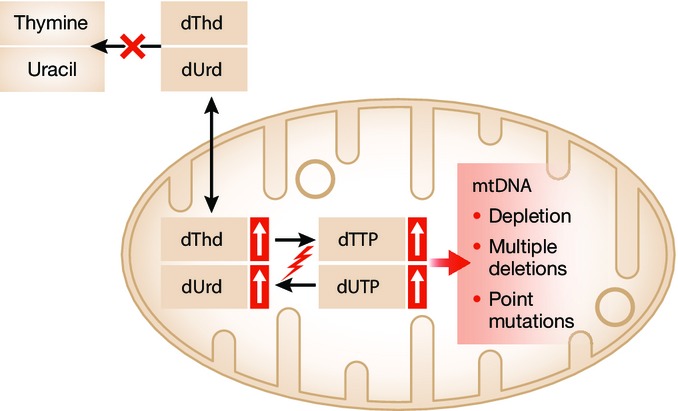
Deoxynucleotide pool imbalance in MNGIE Loss of thymidine phosphorylase (TP) activity causes toxic accumulation of deoxythymidine and deoxyuridine nucleosides and dNTP pool imbalances, which impair mtDNA replication causing depletion, multiple deletions, and point mutations.

As mentioned above, impairment of TP causes systemic accumulation of dThd and dUrd, whose concentrations reach 100-fold higher than normal levels, both in body fluids and tissues (Martí *et al*, 2003), thus perturbing the deoxynucleoside triphosphate (dNTP) pools (Fig 4). A mitochondrial-specific thymidine kinase (TK2) converts deoxynucleosides to their corresponding monophosphates (Bogenhagen & Clayton, 1976). However, while cytosolic thymidine kinase (TK1) is tightly regulated, especially during cell division, mitochondrial TK2 is expressed constitutively (Johansson & Karlsson, 1997), thus contributing to the alterations of the mitochondrial dNTP pool.

This dNTP imbalance interferes with mtDNA replication and results in depletion, multiple deletions, and point mutations, which in turn cause mitochondrial dysfunction. This notion has been demonstrated *in vitro*, where cells exposed to dThd showed unbalanced mitochondrial dNTP pools with increased dTTP and dGTP and decreased dATP and dCTP, causing depletion or multiple deletions of mtDNA (Pontarin *et al*, 2006). Accordingly, in the TP/UP double-knockout (TP^−/−^UP^−/−^) MNGIE model, 4- to 65-fold elevated tissue levels of dThd and dUrd relative to wild-type littermates were found, accompanied by increased dTTP and decreased dCTP in brain mitochondria (López *et al*, 2009). Although this mouse model only recapitulates the biochemical imbalances of the disease and some molecular features in very old animals, it represents the best tool available to investigate the pathomechanisms of MNGIE (Torres-Torronteras *et al*, 2014).

It is important to note that, while mtDNA is sensitive to nucleotide pool imbalance, nuclear DNA (nDNA) appears not to be. This observation can be explained considering how pyrimidine metabolic pathways are used by cells in different cell-cycle states. In quiescent cells, the production of dTTP and dUTP mainly depends on the phosphorylation of dThd and dUrd through the mitochondrial salvage pathway (Pontarin *et al*, 2003; López *et al*, 2009). Nucleotide imbalances interfere with mtDNA but not nDNA replication, because post-mitotic cells do not replicate nDNA, but continuously turnover mitochondria and thus replicate mtDNA. This explanation is also consistent with the observation that the affected tissues in MNGIE are post-mitotic ones. However, in dividing cells, dThd and dUrd triphosphates are generated mainly through the cytosolic *de novo* pathway (Pontarin *et al*, 2003); as a consequence, an excess of nucleosides will not necessarily lead to triphosphate imbalances and thus replication of nDNA or mtDNA will not be affected in proliferating tissues. Interestingly, mouse embryonic fibroblasts from mice carrying extra alleles of the RNR regulatory subunit RRM2 (and therefore with increased RNR activity, which is a rate-limiting step for the production of deoxynucleotides), do not have elevated steady-state dNTP levels, but present reduced chromosomal breakage at fragile sites in nDNA under replication stress stimuli. Therefore, modulation of nucleotide metabolism might be beneficial for nDNA maintenance in the context of replication stress conditions, which reduce dNTP availability (Lopez-Contreras *et al*, 2015).

The accumulation of high levels of dThd and dUrd in the skeletal muscle of patients appears to be an apparent paradox, since muscles express little or virtually no TP. The explanation for this “muscle paradox” is that skeletal muscle is affected not because of TP dysfunction (or lack thereof), but rather because of the toxic effects of accumulated dThd and dUrd (Hirano *et al*, 2006). Indeed, one of the functions of high expression levels of TP in liver, spleen, platelet, lymphocytes, and other tissues is to keep in check the levels of circulating dThd and dUrd, produced by cellular metabolism and turnover of nucleic acids (Nishino *et al*, 1999).

## Therapeutic approaches

Therapeutic strategies aimed at removing or reducing noxious catabolites could potentially improve clinical outcome in EE and MNGIE, and in some cases, the approaches could be similar. Current treatments are most often supportive, and to date, only small controlled clinical trials have been performed, with the primary goal of improving quality of life. There are, however, several exciting strategies at the developmental stage aimed at overcoming the primary defect and alleviate its effects. Some of them have already been tested in cell cultures or animal models and will be described below. The challenge that lies ahead is the translation of the most promising laboratory results into safe and effective therapies for patients.

### Drug-based approaches

The first, simple, and readily applicable strategy is to find molecules that reduce the circulating toxic compound(s), or restore the levels of missing or reduced metabolic intermediates. For instance, in the case of EE, off-label use of common drugs such as N-acetylcysteine (NAC) and metronidazole may reduce H_2_S. N-acetylcysteine (NAC) is a cell-permeable precursor of glutathione (GSH), an abundant compound in mitochondria (Marí *et al*, 2009) where it acts as one of the physiological acceptors of the sulfur atom of H_2_S operated by SQR. Metronidazole is a drug widely used to combat anaerobic infections by reducing the bacterial load in the large intestine (Samuelson, 1999).

Combined exposure to metronidazole and NAC effectively prolongs survival of *Ethe1*^−/−^ mice and also improves the main symptoms in EE patients as shown in a pilot study (Viscomi *et al*, 2010), including marked attenuation or disappearance of the vascular lesions and diarrhea, as well as amelioration of some neurological abnormalities. These encouraging results suggest that the invariably fatal clinical course of EE might be modified by a pharmacological treatment based on the repurposing of low-cost and relatively safe drugs.

Supplementation of deoxycytidine (dCtd) has been shown to prevent mtDNA copy number reduction in a cellular MNGIE model based on thymidine-induced mtDNA depletion. Similar ameliorative effects were obtained by the inhibition of deoxynucleotide catabolism with tetrahydrouridine (THU; inhibitor of cytidine deaminase) or immucillin-H (inhibitor of purine nucleoside phosphorylase) (Cámara *et al*, 2014). In addition, in a MNGIE mouse model (see below), mitochondrial dNTP content can be modulated by systemic administration of dCtd or THU (Cámara *et al*, 2014).

To date, there are no pharmacological treatments available for MNGIE, although drugs aimed at decreasing the rate of renal reabsorption of dThd by chemically inhibiting nucleoside carriers might prove beneficial by decreasing circulating dThd and dUrd levels in MNGIE patients (Lara *et al*, 2007).

### Cell therapy

Replacement of a deficient enzyme in a readily accessible compartment such as blood might prove sufficient to eliminate the toxic compounds in plasma, create a positive diffusion gradient and subsequently clear the freely diffusible toxic compounds from the tissue compartments. Both hemodialysis (Yavuz *et al*, 2007) and platelet infusions (Lara *et al*, 2006) have been tested in unsuccessful attempts to lower the circulating concentrations of thymidine and deoxyuridine. Allogenic hematopoietic stem cell transplantation (AHSCT) has, however, proven to be more successful in ameliorating the clinical course of MNGIE through the normalization of cellular nucleotide pools (Hirano *et al*, 2006; Halter *et al*, 2011). This approach affords sustained correction of enzyme deficiency and has indeed become an established treatment for many different diseases, for instance, purine nucleoside phosphorylase (PNP) deficiency, which biochemically resembles MNGIE since loss-of-function mutations in the PNP gene lead to accumulation of purine nucleosides (Delicou *et al*, 2007).

To date, twelve MNGIE patients have been treated with allogeneic HSCT (Peedikayil *et al*, 2015), with evidence of rapid restoration of enzyme activity together with a reduction or disappearance of plasma dThd and dUrd in patients who engrafted. There remain, however, several limitations: (1) limited tolerance of the patients for transplant-related complications, (2) low engraftment rates, and (3) risk of graft rejection, which mandates for adequate conditioning and immunosuppression. Five patients who had undergone HSCT for MNGIE are still alive, and all demonstrated reduction or disappearance of plasma deoxythymidine and deoxyuridine (Peedikayil *et al*, 2015). An improvement of the gastrointestinal symptoms and a slight amelioration of the neurological signs have been observed in two patients. Nonetheless, more than 70% of transplanted patients died due to the limitations mentioned above (Boschetti *et al*, 2014). Clearly, proper assessment of the effective long-term benefits of bone marrow transplantation in MNGIE requires further experimentation and methodological improvement.

An additional cellular approach for MNGIE is carrier erythrocyte-entrapped deoxythymidine phosphorylase therapy (CEETP) (Moran *et al*, 2008). The encapsulation of therapeutic enzymes within autologous erythrocytes is applicable to disorders where the pathological plasma metabolite is able to permeate the erythrocyte membrane. In a first-in-human experiment, the administration of encapsulated erythrocytes was reported to be effective in reducing/eliminating the elevated plasma and urine concentrations of deoxythymidine and deoxyuridine, although the clinical conditions of patient remained severe leading to premature death for pneumonia 21 days after CEETP (Moran *et al*, 2008).

Based on the same concept, lentiviral-mediated hematopoietic transduced cells were tested in the MNGIE mouse model to restore TP activity in blood cells. Specifically, immunoselected hematopoietic lineage-negative cells from TP/UP double-knockout mice were collected and transduced *in vitro* using a lentivirus-expressing *TYMP* gene and re-infused into partially myeloablated double-knockout mice. Four weeks after transplantation, high TP activities were achieved in peripheral blood cells of treated mice, as compared with undetectable or negligible values in untreated and sham-treated double knockout, followed by reduced plasma dThd and dUrd concentrations to the levels found in wt mice (Torres-Torronteras *et al*, 2011). The limitations of lentiviral vector-based transduction are well known, however, and include possible insertional oncogenesis. In addition, in the absence of a selective advantage of the gene-corrected hematopoietic stem cells, some level of myeloablative conditioning would be required, posing additional risks for the patients.

### AAV-mediated gene therapy

Adeno-associated virus (AAV)-based gene replacement is one of the most promising approaches in the treatment of diseases caused by the accumulation of circulating toxic compounds. In the last decade, AAV-mediated gene delivery has emerged as an effective and safe tool for both pre- clinical and clinical studies of genetic disorders, since the vectors are not pathogenic in humans and mice, remain episomal (thus reducing the risks of integration into the genome), and persist for long time. The existence of AAV serotypes with different tropism and the opportunity to use different tissue-specific promoters make this system a powerful one for the delivery of therapeutic genes in a tissue- and temporal-specific manner (Grieger & Samulski, 2005). Because the liver is the first and foremost detoxifier of potentially harmful circulating compounds, AAV-mediated liver-specific enzyme expression may be beneficial in the treatment of EE and MNGIE, as recently demonstrated in humans affected by hemophilia B (Nathwani *et al*, 2011).

This strategy has been successful in markedly prolonging the survival and restoring biochemical profile of constitutive *Ethe1*^−/−^ mice (Di Meo *et al*, 2012). Intravenous injection of a AAV2/8 vector carrying the *ETHE1* gene under the thyroxine-binding globulin (TBG) promoter at postnatal day 21 (P21), resulted in efficient transduction of hepatocytes (due to the tropism of serotype 8) and liver-specific expression (due to the hepatic promoter TBG), leading to the recovery of enzymatic activity, restoration of the biochemical profile, and marked extension of survival in all treated mice; in fact, most treated animals were alive and well up to 8 months after birth.

The same approach has been applied in the MNGIE mouse model: intravenous injection of AAV2/8 carrying the *TYMP* gene under the TBG promoter resulted in robust and stable TP expression in liver, clearing the systemic accumulation of dThd and dUrd over the time, with no signs of toxicity or hepatocellular damage (Torres-Torronteras *et al*, 2014). The use of hepato-specific AAV in MNGIE is justified also by evidence that liver is an important source of TP (Boschetti *et al*, 2014).

Based on the above, orthotopic liver transplantation may also be considered as a therapeutic option for MNGIE, as well as EE patients. However, the elevated risks connected to the invasiveness of the technique, transplant-related complications, rejection, and overall patient clinical conditions make this approach less safe and feasible than AAV-based gene therapy.

There are thus realistic positive perspectives for the treatment of human EE and MNGIE, especially considering the proof-of-principle, evidence-based demonstration that AAV-driven gene therapy is an effective and feasible approach for the treatment of circulating toxic compounds disorders and which is directly translatable to clinical practice.

## Conclusions

Despite different genetic, molecular, and clinical traits, mitochondrial diseases caused by the accumulation of toxic substances share common features, including possible therapeutic strategies. In principle, the accumulation of a harmful compound can be contrasted more effectively than the intrinsic failure of OXPHOS energy metabolism. The elucidation of the specific toxic mechanisms underpinning disorders such as EE and MNGIE is a fundamental step toward the development of an effective, feasible cure for these fatal mitochondrial diseases in humans.

## Conflict of interest

The authors declare that they have no conflict of interest.

## Pending issues

How does sulfide inhibit SCAD and BCAD?

Why is COX activity normal in the livers of EE mice and patients?

What is the molecular and biochemical basis of tissue-specific vulnerability to deoxythymidine phosphorylase deficiency?

Why does the MNGIE TP/UP double-knockout mouse model not display the clinical signs typical of human disease?

## Abbreviations

Acylcarnitines: Acylated forms of the quaternary ammonium compound carnitine, which allow the translocation of fatty acids from cytosol to mitochondria.
Adeno-Associated Virus (AAV): Small non-enveloped, replication-defective Dependoparvovirus belonging to the Parvoviridae virus family, representing one of the most promising gene therapy vectors.
Antioxidant: Molecules that interact with and neutralize free radicals, thus preventing them from causing damage.
Cytochrome c oxidase (COX): Complex IV, which catalyzes the reduction of oxygen to water, is responsible for the final step in mitochondrial electron transport chain.
Ethylmalonic acid (EMA): Dicarboxylic organic acid produced by the carboxylation of butyrate is a hallmark of enzymatic defects of β-oxidation of fatty acids and branched-chain amino acids.
Gasotransmitter: Small molecular weight endogenous gases with messenger activities and relevant physiological functions. Their production and metabolism are enzymatically regulated, and their effects are not dependent on specific membrane receptors.
Hematopoietic stem cells (HSC): Multipotent, self-renewing progenitor cells that develop from mesodermal hemangioblast cells, and from which all blood cells differentiate.
Intestinal bacterial flora: The normal community of microorganism species residing within the intestinal lumen.
Lactic acidosis: Form of metabolic acidosis due to the accumulation lactic acid, a by-product of anaerobic respiration normally cleared from the blood by the liver, kidney, and skeletal muscle.
Metronidazole: A nitroimidazole anti-infective compound selectively active against anaerobic bacteria and sensitive protozoal organisms. It is a pro-drug activated in anaerobic organisms by the redox enzyme pyruvate-ferredoxin oxidoreductase.
Mitochondrial respiratory chain (MRC): Group of specific carrier complexes located in the inner mitochondrial membrane that transfer electrons from hydrogen to oxygen. Electrochemical energy generated via electron transport is transferred to ADP to reform ATP during the oxidative phosphorylation process.
mtDNA depletion: Reduction in mtDNA copy number, due to impairment of mtDNA maintenance, which causes a heterogeneous group of severe and usually lethal diseases in infancy and childhood
